# Reasons for cannabidiol use: a cross-sectional study of CBD users, focusing on self-perceived stress, anxiety, and sleep problems

**DOI:** 10.1186/s42238-021-00061-5

**Published:** 2021-02-18

**Authors:** Julie Moltke, Chandni Hindocha

**Affiliations:** 1 Clinic Horsted, Chronic Pain Clinic, Farvegade 2, 1463 Copenhagen, Denmark; 2 Clinical Psychopharmacology Unit, Department of Clinical, Educational & Health Psychology, University College London, London, UK; 3 Translational Psychiatry Research Group, Research Department of Mental Health Neuroscience, Division of Psychiatry, Faculty of Brain Sciences, University College London, London, UK; 4 University College Hospital National Institute of Health Research (NIHR) Biomedical Research Centre, London, UK

**Keywords:** Cannabidiol, Stress, Anxiety, Sleep, Internet-survey, Sublingual

## Abstract

**Background:**

Public and medical interest in cannabidiol (CBD) has been rising, and CBD is now available from various sources. Research into the effects of low-dose CBD on outcomes like stress, anxiety, and sleep problems have been scarce, so we conducted an online survey of CBD users to better understand patterns of use, dose, and self-perceived effects of CBD.

**Methods:**

The sample consisted of 387 current or past-CBD users who answered a 20-question online survey. The survey was sent out to CBD users through email databases and social media. Participants reported basic demographics, CBD use patterns, reasons for use, and effects on anxiety, sleep, and stress.

**Results:**

The sample (*N* = 387) consisted of 61.2% females, mostly between 25 and 54 years old (72.2%) and primarily based in the UK (77.4%). The top 4 reasons for using CBD were self-perceived anxiety (42.6%), sleep problems (42.5%), stress (37%), and general health and wellbeing (37%). Fifty-four per cent reported using less than 50 mg CBD daily, and 72.6% used CBD sublingually. Adjusted logistic models show females had lower odds than males of using CBD for general health and wellbeing [OR 0.45, 95% CI 0.30–0.72] and post-workout muscle-soreness [OR 0.46, 95%CI 0.24–0.91] but had higher odds of using CBD for self-perceived anxiety [OR 1.60, 95% CI 0.02–2.49] and insomnia [OR 1.87, 95% CI 1.13–3.11]. Older individuals had lower odds of using CBD for general health and wellbeing, stress, post-workout sore muscles, anxiety, skin conditions, focusing, and sleep but had higher odds of using CBD for pain. Respondents reported that CBD use was effective for stress, sleep problems, and anxiety in those who used the drug for those conditions.

**Conclusion:**

This survey indicated that CBD users take the drug to manage self-perceived anxiety, stress, sleep, and other symptoms, often in low doses, and these patterns vary by demographic characteristics. Further research is required to understand how low doses, representative of the general user, might impact mental health symptoms like stress, anxiety, and sleep problems.

**Supplementary Information:**

The online version contains supplementary material available at 10.1186/s42238-021-00061-5.

## Introduction

In the past years, cannabidiol (CBD), one amongst hundreds of naturally occurring phytocannabinoids found in the *Cannabis sativa* plant, has received a lot of attention from scientific communities, politicians, and mainstream media channels. CBD is the second most abundant cannabinoid in the *Cannabis sativa* plant after delta-9-tetrahydrocannabinol (THC), but unlike THC, CBD is not intoxicating (Pertwee 2008). In many countries, including the UK, there is unsanctioned availability of products containing CBD, from oils and capsules to chewing gums, mints, soft drinks, gummies, and intimate lubrication gels.

CBD has not demonstrated any potential for abuse or dependency and is considered well tolerated with a good safety profile, according to a report released by the World Health Organization (WHO) (Geneva CANNABIDIOL (CBD) n.d.). Since January 2019, the European Union (EU) has classified CBD as a novel food, implying that before 1997, consumption was insignificant. Each country has implemented the regulation of CBD differently. In the UK, The Food Standards Agency (FSA) recommends limiting the daily dose of CBD to 70 mg (Cannabidiol (CBD) n.d.). However, researchers have used doses up to 1200 mg without serious side-effects (Davies and Bhattacharyya 2019). Conversely, few clinical trials involving children with treatment-resistant epilepsy who received either 10 or 20 mg/kg of CBD (Epidiolex) for 12 weeks recorded side-effects, such as a reversible rise in liver enzymes (Devinsky et al. 2018a; Thiele et al. 2018).

The popularity of CBD can be partly explained by an increasing number of preclinical and clinical studies indicating a range of potential health benefits. However, mass media interest also plays a significant role. Studies suggest CBD might help with mental health symptoms and neurological conditions like experimentally induced anxiety (Zuardi et al. 1993), generalised social anxiety disorder (Bergamaschi et al. 2011), social phobia (de Faria et al. 2020), and conditions like PTSD (Elms et al. 2019; Shannon and Opila-Lehman 2016) schizophrenia (Zuardi et al. 2006; Leweke et al. 2012; Morgan and Curran 2008; Schubart et al. 2011), addiction (Hurd et al. 2019; Hindocha et al. 2018; Galaj et al. 2020), and epilepsy (Devinsky et al. 2017; Devinsky et al. 2018b; Cunha et al. 1980). These mental health disorders are often co-morbid and include other symptoms CBD might help with, e.g. sleep and impaired cognition. There is also data to suggest CBD could help treat neurodegenerative diseases like Alzheimer’s disease (Watt and Karl 2017; Fernández-Ruiz et al. 2013; Esposito et al. 2006), Parkinson’s disease (Fernández-Ruiz et al. 2013; García-Arencibia et al. 2007), and chronic pain conditions including fibromyalgia (Van De Donk et al. 2019), either alone or with THC (Rog et al. 2005; Berman et al. 2004; Wade et al. 2003; Svendsen et al. 2004; Notcutt et al. 2004). Additionally, in more than 30 countries, health authorities have approved CBD, under the name Epidiolex, to treat two severe forms of treatment-resistant childhood epilepsy (Dravet and Lennox-Gastaut syndrome) (Devinsky et al. 2016; Silvestro et al. 2019). Sativex, a sublingual spray containing an equal amount of THC and CBD, is also approved to treat multiple sclerosis in more than 30 countries (Keating 2017).

When used in high doses, somnolence is a primary adverse effect (Machado Bergamaschi et al. 2011). Patients in CBD clinical trials were more likely to experience sedation (OR 4.21, 95% CI 1.18–15.01) and somnolence (OR 2.23, 95% CI 1.07–4.64) in comparison to placebo (Chesney et al. 2020). Despite this preclinical and experimental research, there is a lack of human clinical trials to establish the efficacy and appropriate CBD indications fully. The effective dose for most of the above indications is still to be determined. In much of the research, high doses of CBD are used (between 300 and 1200 mg), whilst at the same time, globally, millions of CBD users are using low dose CBD. Thus, a disconnect exists between clinical research and the current state of the market.

A cross-sectional study of 2409 cannabidiol users from the USA found that the top three medical conditions reported were chronic pain, arthritis/joint pain, and anxiety, followed by depression and insomnia (Corroon and Phillips 2018). A recent survey carried out by Wheeler et al. of 340 young adults, some of whom were CBD users, found the top reasons to be stress relief, relaxation, and sleep improvement. They found edible CBD products to be the most prevalent (Wheeler et al. 2020). Another study of 400 CBD patients in New Zealand observed an increase in overall quality of life, a decrease in perceived pain, depression, and anxiety symptoms, as well as an increase in appetite and better sleep (Gulbransen et al. 2020).

A national survey indicated that in the UK, 8–11% of the adult population had tried CBD by June 2019 (Andrew et al. 2019). Studies of Google searches have shown considerable increases in CBD interest, with 6.4 million unique searchers in the USA in April 2019 (Leas et al. 2019). Yet it is clear that scientists, physicians, and governments were not prepared for the rapid surge in CBD use.

The regulatory confusion, along with recent media hype, has made it hard for most people to understand the true nature of CBD. Being classified as both a medicine and a supplement in some forms, whilst an illegal substance in others leads to consumer and patient confusion and potential frustration. Therefore, this study aimed to understand users’ consumption patterns regarding dose, route of administration, and reasons for using CBD. We hypothesised that out of all reasons for using CBD, the top three would be anxiety, sleep disturbances, and stress.

## Methods

We developed an anonymous online questionnaire to collect CBD users’ self-reported characteristics, preferred method/s, and reason/s for using CBD. The survey was hosted on Survey Monkey Inc. (San Mateo, CA, USA). Data was collected between 10 January 2020 and 18 March 2020. The 20 questions were designed as multiple-choice questions with the option to choose either one or more answers. For some questions, respondents could write an alternative response if no option matched. We collected demographic information (age, sex, and location), CBD use patterns, reasons for use, other medication use, perceived effects, and side effects. The full questionnaire is provided in the supplementary materials.

To access actual CBD users, we collaborated with four different CBD brands and retailers (TheDrug.Store, OTO CBD, With Pollen and Grass & Co.), based in the UK, who sent out the survey to their email databases. The survey was sent out to 14,743 unique email addresses. Two thousand five hundred thirty-four were opened and 475 clicked through to the survey. We also shared the survey with CBD user groups on social media channels like Facebook and LinkedIn. We did not collect any personal data or IP addresses. Ethical approval was not required since this research investigated non-sensitive information using anonymous survey procedures with participants not defined as “vulnerable”. In addition, participation was deemed unlikely to induce undue psychological stress or anxiety based on ethics committee guidelines (UCL REC n.d.).

### Statistical analysis

All analyses were conducted in SPSS version 24 (IBM Corporation, Armonk, NY). Valid percentages are reported rather than absolute values for descriptive statistics to account for missing data. We only report data on those reporting using CBD themselves equivalent to 90% of the respondents (e.g., not for veterinary use, not those who had not tried it, and those reporting on behalf of other users). An analysis of non-responders can be found in supplementary materials. We conducted logistic regression models to investigate associations between sex (males [reference category] and females), age (recoded to < 34 years old [reference category], between 35 and 54 years old, and 55+) and location (UK [reference category], other). For CBD use patterns, we used separate models to compare those who did and did not report their primary use of CBD for self-perceived anxiety, stress, and sleep whilst controlling for sex, age, and location. We dummy-coded “time of day” as each category versus all others. We report adjusted odds ratios with 95% confidence intervals and *p* values with a defined cut-off of 0.05.

## Results

**Fig. 1:**
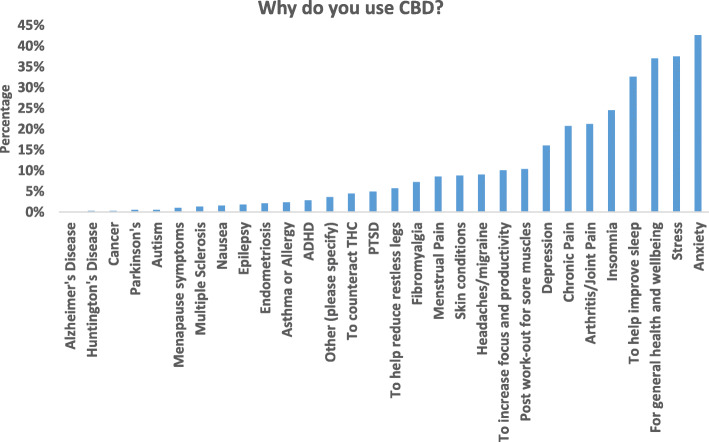
Reasons for cannabidiol use amongst 397 adult cannabidiol users who were allowed to respond to more than one option leading to a total of 1622 responses. *Y*-axis represents percentage based on total responses

**Fig. 2:**
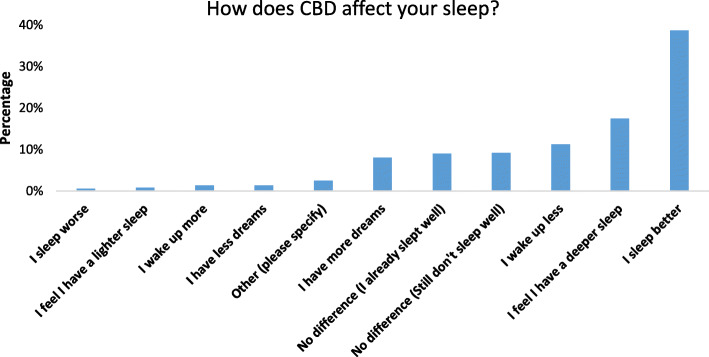
Perceived effects of cannabidiol on sleep amongst adult cannabidiol users responding to the question “how does cannabidiol affect your sleep?” Participants were allowed to select multiple options. *Y*-axis represents percentage of total responses (*n* = 522)

**Fig. 3:**
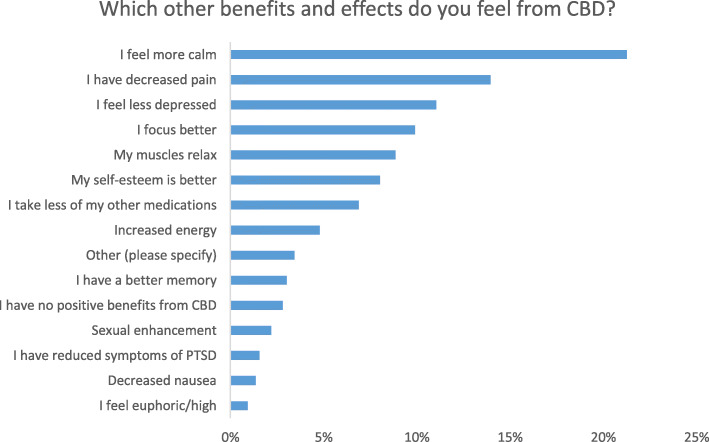
Other perceived benefits of cannabidiol amongst adult cannabidiol users. Respondents were asked what other benefits or effects they feel from using cannabidiol. Participants were allowed to select multiple options. *X*-axis is the percentage of total responses (*n* = 906)

The most significant findings were that many CBD users reported that CBD could improve sleep problems, stress, and anxiety and be used for general health and wellbeing. In the detailed results below, you can find the demographics of our survey population (Table 1), the CBD use patterns (Table 2), and logistic regression and OR’s for the different subgroups. The indications for CBD use are shown (Fig. 1), as well as how CBD affects sleep (Fig. 2), and other effects of CBD (Fig. 3). Using CBD for sleep was associated with taking it in the evening, and using CBD for anxiety or stress was associated with the sublingual route. Females had higher odds of using CBD for anxiety and men for post-workout. Details of the results can be found below.

**Table 1:** Demographic characteristics of 387 adult cannabidiol users

	*N* (total *N* = 387)	Valid percentage (%)
Sex (% F)	237	61.2
Age in years (*n* = 385)		
18–24	16	3.9
25–34	92	23.9
35–44	96	24.9
45–54	90	23.4
55–64	55	14.3
65+	36	9.4
Country (*n* = 380)		
UK	294	77.4
USA	31	8.1
Denmark	21	5.5
Other	34	8.9
CBD purchase location (*n* = 382)		
Online CBD shop (legal)	265	69.37
Health shop	44	11.5
Pharmacy	14	3.7
Darkweb	2	0.5
From a health provider who sells CBD	6	1.6
Medical prescriber (physician)	2	0.5
I make it myself	11	2.9
Other	38	10

**Table 2:** Patterns of cannabidiol use in 287 cannabidiol users

	*N* (%)
**Length of CBD use (*n*** **= 387)**	
0–3 months	8 (2.1%)
3–6 months	145 (37.7%)
6–12 months	115 (29.9%)
1–2 years	64 (16.6%)
2–5 years	37 (9.6%)
More than 5 years	16 (4.1%)
**Time of day used (*n*** **= 387)**	
Morning	70 (18.1%)
Evening	99 (25.6%)
Morning and evening	92 (23.8%)
Multiples times per day (> 2)	46 (11.9%)
When needed	78 (20.2%)
Other	2 (0.5%)
**Routes of administration (*n*** **= 387)**	
Sublingual	281 (72.6%)
Capsules/pills	68 (17.6%)
Topical (on skin)	54 (14%)
Vaping	36 (9.3%)
Edibles	33 (8.5%)
Drinking	26 (6.7%)
Spray (via mouth)	20 (5.2%)
Smoking	18 (4.7%)
Suppository (vaginal/rectal)	3 (0.8%)
Other	13 (3.4%)
**Dose of CBD per day in mg (*n*** **= 373)**	
0–24	110 (29.5%)
25–49	93 (24.9%)
50–99	69 (18.5%)
100–149	25 (6.7%)
150–199	11 (2.9%)
≥ 200	27 (7.24%)
I do not know	38 (10.2%)

### Demographic characteristics

A total of 430 people started the survey, of whom 15 (3.48%) did not respond to any questions, and 28 (6.5%) reported they did not use CBD themselves (analysis of these non-users can be found in the supplementary materials). Non-CBD-users skipped most questions and had sociodemographic characteristics similar to those of CBD users. Three hundred eighty-seven (90%) reported using CBD themselves. The majority of users were females from the UK (see Table 1). In regards to other medication use, there were a total of 467 responses. 39.4% of respondents reported not taking any other medication, 14.7% “painkillers”, and 14.7% “other” (40% anxiolytics and antidepressants). No other medication was reported by more than 10% of responses.

Logistic regression on location purchased (CBD shop or other) found that those who lived outside of the UK (aOR 2.286, [95% CI 1.35–3.86], *p* = 0.002) and males (aOR 1.75, [95% CI 1.06–2.88], *p* = 0.02) had greater odds of purchasing CBD from an “other” location. Each of the primary disorders was included in the model individually, and did not significantly alter the model and were not associated with location purchased.

### CBD use patterns

The majority of users take CBD sublingually for 3–6 months (see Table 2). Those 35–54 years old (aOR 1.67 [95% CI 1.02–2.72], *p* = 0.04) and those 55+ (aOR 2.01, [95% CI 1.11–3.64], *p* = 0.02) had greater odds of using CBD daily in comparison to less than daily. There were no associations with self-perceived anxiety, stress, or sleep improvement. Females had lower odds of using CBD for greater than 1 year versus less than 1 year (aOR 0.54, [95% CI 0.38–0.88], *p* = 0.013) suggesting females had used CBD for less time. No associations emerged for self-perceived anxiety, stress, or sleep. There were no sex or age associations for the frequency of use, duration of use, or number of times per day. Females had a greater odds of responding that they take CBD when they need it versus males (aOR 1.79, [95% CI 1.036–3.095], *p* = 0.037). However, no other associations with age and sex on time of day emerged.

Compared with people not using CBD for anxiety, those who did self-medicate used CBD multiple times a day (aOR 3.44, [95% CI 1.70, 7.00], *p* = 0.001). Moreover, compared with those not using CBD for self-perceived stress, those who were self-medicating also used CBD multiple times a day (aOR 1.97, [95% CI 1.034–3.77], *p* = 0.039). Those using CBD for sleep improvement had greater odds of using CBD in the evening (aOR 3.02, [95% CI 1.86, 4.93], *p* ≤ 0.001) and lower odds of using CBD in the morning (aOR 0.157, [95% CI 0.07–0.38], *p* ≤ 0.001). Those using CBD for self-perceived anxiety had lower odds of using CBD in the evening (aOR 0.56, [95% CI 0.14–0.45], *p* ≤ 0.001). No associations emerged between those who did and did not use CBD for self-perceived stress on the time of day they used CBD.

### CBD dose and route of administration

Route of administration did not vary by sex. There were lower odds of those aged 55+ of vaping CBD (aOR 0.176, [95% CI 0.04–0.80], *p* = 0.025) as well as lower odds of those aged 35–55 (aOR 0.245, [95% CI 0.10–0.59], *p* = 0.002) and 55+ (aOR 0.115, [95% CI 0.025–0.520], *p* = 0.005) in comparison to 18–34 years old for drinking CBD. Self-reported anxiety (aOR 1.78, [95% CI 1.08–2.92], *p* = 0.023) and those using CBD for sleep improvement (aOR 1.945, [95% CI 1.152–3.285], *p* = 0.013) were associated with the sublingual route. Stress was not associated with route of administration.

### Reasons for use of CBD

42.6% endorsed using CBD for self-perceived anxiety, followed by 37.5% for stress, 37% for general health and wellbeing, and 37% for improving sleep (see Fig. 1). 24.6% reported use for self-perceived insomnia. Overall, 42.5% of respondents said they were using CBD for some sleep issue, either to improve sleep or for self-perceived insomnia. In the supplementary materials (see Table 2), we show reasons for use broken down by sex, age, and location.

In adjusted logistic models, more males (47.4%) were using CBD for general health and wellbeing than females (30.7%; aOR 0.464, [95% CI 0.30–0.72], *p* = 0.001). More females were using CBD for self-perceived anxiety (47.9%) than males (34.2%; aOR 1.595, [95% CI 1.021, 2.49], *p* = 0.04), and for self-perceived insomnia (females 28.6%, males 17.8%; aOR 1.871, [95% CI 1.125–3.112], *p* = 0.015). More males (14.1%) than females (7.1%) were using CBD for post-workout sore muscles (aOR 0.462, [95% CI 0.236–0.905], *p* = 0.024).

Those aged 35–54 years old (33.9%; aOR 0.60, [95% CI 0.363–0.995], *p* = 0.048) and 55+ (31.9%; aOR 0.526, [95% CI 0.287–0.964], *p* = 0.038) had lower odds of using CBD for general health and wellbeing than those aged 18–34 years old (47.7%). Moreover, those aged 35–54 years old (37.1%, aOR 0.561, [95% CI 0.346–0.911], *p* = 0.019) and 55+ (20.9%; aOR 0.249, [95% CI 0.132–0.470], *p* ≤ 0.001) had lower odds of using CBD for stress versus those aged 18–34 years old (51.4%). Those aged 34–55 (24.8%) had greater odds of using CBD for chronic pain versus 18–34 years old (14.7%; aOR 2.093, [95% CI 1.122–3.905], *p* = 0.02). Those aged 55+ (5.1%) had lower odds of using CBD for post-workout sore muscles than 18–34 years old (15.5%; aOR 0.302, [95% CI 0.105–0.868], *p* = 0.026). Those aged 35–54 years old (43.5%; aOR 0.464, 95% CI 0.28–0.76, *p* = 0.002) and 55+ (19.8%; aOR 0.149, [95% CI = 0.077, 0.289], *p* < 0.001) had lower odds of using CBD for self-perceived anxiety versus 18–34 years old (60.4%). Use of CBD for arthritic/joint pain was higher in those 55+ (41.8%) (aOR 8.569, 95% CI [3.792–19.363], *p* < 0.001) and 35–54 years old (16.7%; aOR 2.295, [95% CI 1.041, 5.061], *p* = 0.04) in comparison to those 18–34 years old. Those aged 34–55 years old (5.9%) had lower odds of using CBD for skin conditions than those aged 18–34 years old (14.7% aOR 0.42, [95% CI 0.10–0.93], *p* = 0.03) and those aged 55+ (4.4%) had lower odds of using CBD to improve focus than those aged 18–34 years old (15.3%; aOR 0.248, [95% CI 0.08–0.77], *p* = 0.017). Moreover, those aged 55+ (23.1% versus 18–34 years old: 41.4%) had lower odds of using CBD for sleep improvement (aOR 0.4, [95% CI 0.21, 0.75], *p* = 0.004). For endometriosis and menstrual problems, we removed sex from the model finding those aged 34–55 had lower odds of using CBD for menstrual problems (aOR 0.379, [95% CI 0.18–0.796], *p* = 0.01). Endometriosis did not vary by age. Depression, PTSD, fibromyalgia, ADHD, headache, asthma, THC counteract effects, and restless legs did not vary by sex or age. Confidence intervals could not be generated for Parkinson’s disease, Alzheimer’s disease, autism, multiple sclerosis, epilepsy, cancer, and nausea due to small sample sizes.

### Self-perceived anxiety

One hundred sixty-five of 387 (42.6%) endorsed using CBD for self-perceived anxiety. In response to the question “how does CBD affect your anxiety levels”, participants responded that they felt less anxious (141/163 (86.5%)), followed by “no difference (I still suffer from the same degree of anxiety)” (21/163; 12.8%), and one person (0.6%) noted greater anxiety. Moreover, participants were asked how often they thought about problems when they were supposed to be relaxing, compared with before they started taking CBD. We found that just 96/163 (58.9%) of respondents thought about their problems less than before, followed by “it hasn’t changed (I still think a lot about problems” (55/163; 33.7%), followed by “it hasn’t changed (I did not think about problems a lot before)” (11/163; 6.7%), followed by (1/163; 0.6%) of respondents reporting thinking about problems more than before.

Amongst those who reported experiencing anxiety, adjusted logistic models comparing those who responded that CBD reduces their self-perceived anxiety with those who responded that they still suffer from anxiety found no associations with age, sex, or location. Similar results emerged for “thinking about problems”.

### Self-perceived stress

One hundred forty-five of 387 (37.5%) of respondents endorsed the use of CBD for self-perceived stress. Amongst those using CBD for stress, in response to the question “how does CBD affect your stress level”, participants responded that they felt less stressed (130/141; 92.2% followed by it does not affect my stress levels (I still feel stressed) (11/141; 7.8%). No respondent said that it increased their stress level. Adjusted logistic models comparing those who responded that CBD reduces their stress versus those who responded that they still have stress found no associations with age, sex, or location.

### Self-perceived sleep problems

As we initially designed the study to address sleep, we asked detailed questions regarding this. Improving sleep (125/387; 32.3%) and self-perceived insomnia (95/387; 24.5%) were the fourth and fifth-ranked endorsed reasons for using CBD, overall 42.5% endorsed sleep as a reason for use. Respondents said that CBD helped them sleep (see Fig. 2). As we restricted this analysis to respondents who selected using CBD for sleep improvement, there was considerable overlap between using CBD for sleep improvement and self-perceived insomnia. Regarding questions about the time it takes to fall asleep, 48.2%(73/124;) said CBD led them to fall asleep faster, followed by 29/124 (23.4%) who said it did not make a difference and still have a hard time falling asleep, followed by 22/124 (17.7%) who said it did not make a difference because they did not have a problem falling asleep beforehand. Age, sex, and location were not associated with the speed of falling asleep.

### Other reported benefits

We asked participants to report on other effects they experience. From a total of 960 responses, the most prevalent effect was calm (21.3%), followed by decreased pain (19.5%) (see Fig. 3). One per cent reported feeling euphoric/high. In examining the “other” responses, 27/960 (9.3%) reported that they did not feel any benefits from the use of CBD.

Sex was associated with sexual enhancement where males reported experiencing more sexual enhancement (9.9%) than females (2.9%) (aOR 0.274, [95% CI 0.11–0.70], *p* = 0.007). There were no other associations between sex and other CBD benefits. Those aged 55+ (23.1%; aOR 3.8, [95% CI 1.63–8.87], *p* = 0.002) and those aged 35–54 years old (16.8%; aOR 2.72, [95% CI 1.258–5.876], *p* = 0.011) reported taking less of their other medications in comparison to those aged under 34 years old (9.9%). Those ages 55+ reported experiencing more “no positive experiences” (14.3%) in comparison to those under 34 (2.7%; aOR 5.31, [95% CI 1.45–19.41], *p* = 0.012).

### CBD side-effects

A total of 388 responses were made, of whom 277/388 (71%) were logged as not experiencing any side-effects. Dry mouth was experienced by 44/388 (11%), and 13/288 (3%) experienced fatigue. All other side-effects were reported less than 2% (e.g. dizziness, nausea, upset stomach, rapid heartbeat, diarrhoea, headache, anxiety, psychotic symptoms, sexual problems, trouble concentrating). No respondents reported vomiting, fainting, liver problems (raised liver enzymes in blood test), or seizures. Adjusted logistic models show no associations of age, of sex with “no side effects” or fatigue. Location of the participants was associated with dry mouth, those who lived outside of the UK had greater odds of experiencing dry mouth (aOR 2.44, [95% CI 1.25–4.75], *p* = 0.009). No other side-effects were analysed due to the small number of respondents citing other side-effects.

## Discussion

This study aimed to investigate CBD use patterns in the general population regarding the route of administration, dose, and indications for use. We found that the main indications for using CBD were self-perceived anxiety, stress, general health and wellbeing, sleep, and pain.

### User characteristics and reason for use

More than half of the users were using a daily dose below 50 mg via a sublingual route of administration. Most were using CBD daily, sometimes multiple times per day. We found that respondents who use CBD for self-perceived anxiety and stress tend to use it several times per day, whilst respondents endorsing using CBD for sleep take it in the evening, indicating that user patterns vary according to the symptoms. A recent review suggests half-life is between 1.4 and 10.9 h after oromucosal spray and 2–5 days after chronic oral administration (Iffland and Grotenhermen 2017). In the light of these findings, it may be that people are dosing CBD several times per day to maintain stable plasma levels throughout the day if managing symptoms of stress and anxiety, whilst only using CBD at night if managing sleep problems.

We found that 69.7% of users had been using CBD for less than 1 year. Moreover, only 4.1% had used CBD for more than 5 years, reflecting both that it is a fairly new phenomenon and an increasing interest in CBD in the UK, compared with the USA. A similar American survey reported that 34.6% had used CBD for less than 1 year and 53.2% more than 5 years (Corroon and Phillips 2018). At the time of writing, CBD is legal in all but three, out of fifty, American states, and many of these allow the products to contain THC. In the UK and Europe, non-prescription CBD products are not allowed to contain any THC (< 0.01%). These differences might create a divergence between European vs American consumers’ experiences, and stresses the urgency for internal and external regulation, and education about cannabinoids in Europe.

We found age and sex differences in the reason for CBD use. Most of the sample was female, but males had greater odds of using CBD for general health and wellbeing and post-workout for sore muscles. In contrast, females were more likely to use CBD for self-perceived anxiety and insomnia, reflecting the higher prevalence of both symptoms amongst women (McLean et al. 2011; Li et al. 2002). We also found more females using CBD for fibromyalgia, possibly reflecting the much higher prevalence of fibromyalgia amongst women (Yunus 2002). A recent study compared the subjective effects of 100 mg oral versus vaporised and smoked CBD and found that women reported experiencing more subjective effects of CBD than men (Spindle et al. 2020), which may reflect why women were using CBD for more chronic symptomology. There were also significant age differences, with more people under 34 years old using CBD for general health and wellbeing than older age groups, which might be explained in part by the fact that disease burden generally increases with age. More young people use CBD to reduce self-perceived stress and anxiety, aligning with studies finding young people are more troubled by symptoms of anxiety than older people (Brenes et al. 2008).

In the present study, we found that the largest proportion of respondents used CBD to help with mental health symptoms like perceived anxiety, stress, and sleep problems. This finding aligns with a previous CBD survey that found that anxiety and insomnia were amongst the top 6 reasons for using CBD (Corroon and Phillips 2018). However, Corroon et al. found that the two main reasons for using CBD was arthritis/joint pain and chronic pain, whereas these ranked number six and seven amongst reasons from our respondents. This result may reflect the younger demographics of our sample compared with Corroon et al.

With few variations, the reasons for use in our study were somewhat similar to the results from another study of 400 patients in New Zealand, who were all prescribed sublingual CBD oil with doses ranging from 40 to 300 mg/day (Gulbransen et al. 2020). This study found that the patients had an increase in overall quality of life, including improved sleep and decreased self-perceived anxiety levels and reduced pain scores.

### Route of administration, dosing, and side-effects

Younger respondents were more likely to use novel routes of administration, e.g., vaping or drinking. This trend correlates with data showing that more people have tried vaping (in general) amongst younger age groups (Vaping and e-cigarette use by age U.S 2018). Only 9.3% reported vaping CBD in our sample, compared with 19% in the study by Corroon et al*. (*Corroon and Phillips 2018*)*. The fast onset of vaporised CBD might explain why inhaled CBD is popular for self-perceived anxiety and stress.

Corroon et al. found a more even distribution between various application methods with the most popular being sublingual CBD (23% vs 72,6% in our study sample). Our approach of recruiting respondents through email databases of non-vape CBD brands may explain why the sublingual administration route is much more frequent in our study than in the American survey.

The bioavailability of CBD varies by route of administration (Millar et al. 2019), but is generally low, between 10 and 31% (Millar et al. 2018). Oral routes have the lowest bioavailability due to first-pass metabolism, whilst inhaled routes have the highest bioavailability (Ohlsson et al. 1986). The bioavailability of sublingual CBD is between 13 and 19% (Mechoulam et al. 2002), and greater than the oral route, thus exerting effects at much lower doses, making it more efficient for users. Investigating plasma levels of low-dose sublingual CBD users, and correlating them to the subjective experience, might give important insights into the optimal dose for treating these low-level mental health problems like self-perceived stress, anxiety, and sleep problems.

Most people were using less than 100 mg (72.9%) per day. Due to the high price and the lack of medical supervision, it is not surprising that non-medical CBD users are taking much lower doses than those used in clinical studies, and those prescribed for specific medical conditions (Davies and Bhattacharyya 2019; Szaflarski et al. 2018). It is important to highlight that 16.8% reported using more than 100 mg per day, and 10.2% did not know how much CBD they were using. The use of high doses CBD is concerning in light of the current FSA recommendation of restricting the dose to 70 mg CBD per day (Cannabidiol (CBD) n.d.), and it stresses the importance of better public information and communication and improved packaging and guidance from brands to consumers.

Amongst our study sample, almost 11% experienced having a dry mouth, most likely indicating levels of THC in the product, as this is a common side effect of THC rather than CBD (Darling and Arendorf 1993; LaFrance et al. 2020). People living outside of the UK had higher odds of experiencing a dry mouth, which might be explained by the different legislation regarding permitted THC content and CBD quality between countries. This differentiation leads to some concerns about product safety, consistency, and ultimately trust amongst CBD consumers. A recent study of 29 CBD products showed that only 11% contained within 10% of the advertised CBD concentration, 55% of the products had traces of controlled substances such as THC (Liebling et al. 2020). There is still a need for external and internal control within the CBD industry to ensure consumer safety is prioritised.

### CBD and self-perceived stress

37.5% of respondents reported using CBD for perceived stress, with 92.2% reporting reduced stress levels, making it the third-highest ranking reason for CBD use amongst our sample. Yet, no studies are looking directly at how CBD affects perceived stress levels. This might in part be because stress, apart from post-traumatic stress disorder, is not classified as a disease according to international disease classification (WHO | Burn-out an “occupational phenomenon”: International Classification of Diseases 2019). With more than 12.8 million working days lost because of work-related stress, anxiety, or depression in the UK (Hse 2019), the relationship between CBD and stress is an area of interest for further research. A recent study surveying social media for comments about perceived therapeutic effects of CBD products revealed that the most frequently discussed symptoms, which are not addressed in the research literature, are indeed stress and nausea (Tran and Kavuluru 2020).

### CBD and self-perceived anxiety

Self-perceived anxiety was the top-ranked reason for the use of CBD with 42.6% reporting they take CBD for this reason. Of these, 86.5% reported they felt less anxiety. There are biologically plausible reasons for the use of CBD in anxiety. Pharmacological research suggests CBD is a partial 5-HT1a receptor agonist which supports anxiolytic and stress-reducing properties (Russo et al. 2005; Resstel et al. 2009), the activation of which has been associated with anxiolytic, antidepressant, and antipsychotic effects (Zuardi et al. 1993; Bergamaschi et al. 2011; de Faria et al. 2020; Vilazodone for major depressive disorder | MDedge Psychiatry n.d.; Newman-Tancredi and Kleven 2011). CBD also modulates specifically configured GABA_A_ receptors that may be relevant to anxiolytic effects (Bakas et al. 2017; Deshpande et al. 2011). CBD is anxiolytic under experimental conditions in animals, healthy humans and in those with generalised social anxiety disorder (de Faria et al. 2020; Elms et al. 2019; Newman-Tancredi and Kleven 2011) although large clinical trials have not been conducted. Crippa et al. administered an oral dose of 400 mg CBD or placebo, in a double-blind procedure. They found it significantly lowered feelings of anxiety, accompanying changes in limbic areas, in subjects with social anxiety disorder (SAD) (Crippa et al. 2011). Similar results were seen in a small randomised trial using a public speaking test with 600 mg CBD vs placebo (Bergamaschi et al. 2011).

### CBD and self-perceived sleep problems

In our survey, sleep was the second-highest-ranking reason for CBD use. We found that 42.5% used CBD to help with sleep, which is higher than for previously published data on adult CBD users, where it was the fifth-highest reason (Corroon and Phillips 2018). It is well-known that a lack of sleep can cause a variety of physical and mental health effects including raised levels of cortisol(Leproult et al. 1997), anxiety (Babson et al. 2010), and mood disturbances (Brazeau et al. 2010), and both short and long duration of sleep is a significant predictor of death (Cappuccio et al. 2010). A recent controlled study of 300 mg CBD found no effect on any sleep indices (Linares et al. 2018), whilst observational and cross-sectional studies showed improvement in sleep outcomes (Corroon and Phillips 2018; Gulbransen et al. 2020). Preclinical studies have shown mixed results with some doses showing an increase in total sleep time (Chagas et al. 2013) and another study indicating that CBD causes increased wakefulness (Murillo-Rodríguez et al. 2006). Thus, the research on CBD and sleep thus far is mixed. However, as sedation and somnolence are regarded as common adverse effects of CBD in a meta-analysis of clinical trials where high doses are used (Chesney et al. 2020), it may not be surprising that CBD at low doses improved sleep quality and duration.

Given the low quality of CBD available on the market, it may be that these individuals were not taking CBD, or that CBD is not efficacious in sleep, so many individuals report better sleep by virtue of the placebo effect, fuelled by marketing (Haney 2020). Another reason may be that CBD is acting on other aspects of stress and anxiety that indirectly reduce sleep problems. Still, in this survey, participants directly attributed improved sleep to CBD. This points to the need for RCTs, as the effect of expectations (i.e. the result of the placebo effect), particularly with compounds advertised as cure-alls (Haney 2020). Suggesting that the placebo effect may contribute to the purported impact of CBD does not reject the potential medical value of CBD, but it does mean we must be wary of the results of observational studies (Haney 2020).

### Strengths and limitations

Our measures were retrospective self-reported symptoms, rather than contemporaneous reports or object assessments, and thus prone to recall bias. This approach may lead to over- or under-estimation of benefits and harms. In reporting anxiety symptoms, it should be noted that many anxiety measures are self-reported, and scales are often an accurate measure of anxiety. Stress itself is not often measured, but scales assessing self-reported stress are reliable (Morgan et al. 2014). Regarding sleep problems, our measures do not accurately correspond with objective measures of sleep such as actigraphy (Girschik et al. 2012), which has implications in the epidemiology of sleep, including in the present study. Future research should use validated measures of anxiety, stress, and sleep. However, it should be noted we included responses to gain an insight where CBD may not help, with about 20% responding that CBD did not help with sleep or anxiety and about 10% saying CBD did not help with stress. There is also a risk of selection biases regarding our recruitment method from email databases of current users and social media recruiting. As we had a self-selected sample, we do not represent the general population or even the overall population of CBD users. It is more likely that respondents with a positive experience have responded to this survey, and continue to use CBD. Still, users with a negative experience may have stopped using CBD and therefore were not reached by this survey, which might further contribute to the selection biases.

## Conclusion

The survey demonstrated that CBD is used for a wide range of physical and mental health symptoms and improved general health and wellbeing. A majority of the sample surveyed in this study found that CBD helped their symptoms, and they often used doses below 50 mg. Out of the four most common symptoms, three were related to mental health. Self-perceived stress, anxiety, and sleep problems constitute some of society's biggest health problems, but we lack adequate treatment options. Further research is needed into whether CBD can efficiently and safely help treat these symptoms.

## Supplementary Information

**Additional file 1: **User Survey. **Table S1.** Demographic variables of the 28 non-responders. **Table S2.** Reasons for use of cannabidiol by sex, age and location. Results are presented as n (%).

## Data Availability

The datasets used and analysed during the current study are available from the corresponding author on reasonable request.

## Abbreviations

CBD: Cannabidiol
THC: Delta-9-tetrahydrocannabinol
WHO: World Health Organization
