# Supplementary material for: Reasons for cannabidiol use: a cross-sectional study of CBD users, focusing on self-perceived stress, anxiety, and sleep problems
Source: J Cannabis Res. 2021 Feb 18;3:5. doi: 10.1186/s42238-021-00061-5 (PMC7893882; doi:10.1186/s42238-021-00061-5)
Supplement: Supplementary file 1 — Additional file 1: User Survey. Table S1. Demographic variables of the 28 non-responders. Table S2. Reasons for use of cannabidiol by sex, age and location. Results are presented as n (%). [file 42238_2021_61_MOESM1_ESM.zip › Supplementary materials.1.docx]

**Title:** Reasons for cannabidiol use: A cross-sectional study of CBD users, focusing on self-perceived stress, anxiety, and sleep problems

**Julie Moltke^1^*, Chandni Hindocha^2,3,4^**

**^1^** Clinic Horsted by Tina Horsted, Chronic Pain and Medical clinic, Farvegade 2, 1463 Copenhagen, Denmark

^2^ Clinical Psychopharmacology Unit, Department of Clinical, Educational & Health Psychology, University College London, Faculty of Brain Sciences, University College London

^3^ Translational Psychiatry Research Group, Research Department of Mental Health Neuroscience, Division of Psychiatry, Faculty of Brain Sciences, University College London

^4^ University College Hospital National Institute of Health Research (NIHR) Biomedical Research Centre

* Author to whom correspondence should be addressed: Julie Moltke, drjuliemoltke@gmail.com, Clinic Horsted, Chronic Pain and Medical clinic, Farvegade 2, 1463 Copenhagen, Denmark

**Short title**: Self-reported effects of CBD on mental health outcomes.

**Word count:**

Abstract: 325

Main text: 5564

**Supplementary materials: User Survey**

**CBD user study to investigate self-reported benefits and side-effects.**

Created by medical doctor Julie Moltke, cannabis specialist and editor in chief of the online health and

wellness publication www.getthedose.com.

The aim is to better understand user habits and self-reported benefits of high street CBD. By

completing and submitting this survey, you are indicating your consent to participate in the

questionnaire. We will do our utmost to keep your data confidential. We do not collect IP addresses. If

you have any questions, please email julie@getthedose.com.

All results are anonymous and will be published during spring 2020.

Thank you for your time.

1. Gender

Male

Female

2. Age

Under 18

18-24

25-34

35-44

45-54

55-64

65+

3. How often do you use CBD?

Multiple times per day

One time per day

Multiple times per week

Weekly

Monthly

I don't use CBD

4. At what time of the day do you take CBD?

In the morning

In the evening

In the morning and the evening

Multiple times per day (more than two)

I take it when I need it

Other (please specify)

5. What is the reason for using CBD? (Choose all that apply)

For general health and wellbeing

Stress

Chronic Pain

Anxiety

Insomnia

Arthritis/Joint Pain

Depression

PTSD

Menstrual Pain

Endometriosis

Fibromyalgia

Parkinson's

Alzheimer's

ADHD

Autism

Multiple Sclerosis

Huntington's

Epilepsy

Skin conditions

Headaches/migraine

Asthma or Allergy

Cancer

Nausea

To counteract THC

To increase focus and productivity

To help improve sleep

Post work-out for sore muscles

To help reduce restless legs

Other (please specify)

6. Do you take other medications? (Choose all that apply)

I don't take other medications

Pain-killers (non-opioids)

Pain-killers (opioids)

Heart medicine

Asthma medicine

Other lung medicine

Cancer treatment

Thyroid medicine

Diabetes medicine

Sleeping aid

Other (please specify)

7. How much CBD do you usually take per dose?

0-24 mg

25-49 mg

50-99 mg

100-149 mg

150-200 mg

> 200 mg

I don't know

8. How much CBD do you use per day?

0-24 mg

25-49 mg

50-99 mg

100-149 mg

150-199 mg

200-249 mg

250-299 mg

300-349 mg

350-400 mg

> 400 mg

I don't know

9. How do you usually take CBD? (Choose all that apply)

Sublingually (under the tongue)

Capsules or Pills

Sprayed in the mouth

Topical on the skin

Vaping

Smoking

Suppository (vaginal/rectal)

Edibles

Drinking

Other (please specify)

10. How long have you been using CBD for?

Less than 6 months

6-12 months

1-2 years

2-5 years

More than 5 years

Less than 3 weeks

11. Stress: How has CBD affected your stress level?

It makes me feel less stressed

It makes me feel more stressed

It does not affect my stress level (I still feel stressed)

It does not affect my stress level (I did not feel stressed before)

12. Compared to before you started taking CBD, how often do you find yourself thinking about problems even

when you are supposed to be relaxing?

I think about problems less than before

I think about problems more than before

It hasn't changed (I still think about problems a lot)

It hasn't changed (I did not think about problems a lot before)

13. Anxiety: How does CBD affect your anxiety levels?

I feel less anxious

I feel more anxious

I feel no difference (I still suffer from the same degree of anxiety)

I feel no difference (I do not suffer from anxiety)

14. Sleep: How does CBD affect your sleep? (choose all that apply)

I sleep better

I sleep worse

I wake up less

I wake up more

I feel I have a deeper sleep

I feel I have a lighter sleep

I have more dreams

I have less dreams

I Feel no difference (I still don't sleep well)

I Feel no difference (I already slept well)

Other (please specify)

15. Has the time it takes you to fall asleep changed since you started taking CBD?

I fall asleep faster

It takes me longer to fall asleep

It does not make a difference (I still have a hard time falling asleep)

It does not make a difference (I did not have a hard time falling asleep before)

16. Which other benefits and effects do you feel from CBD? (choose all that apply)

I feel less depressed

I take less of my other medications

My self-esteem is better

I have decreased pain

I have a better memory

I focus better

I have reduced symptoms of PTSD

I feel more calm

Sexual enhancement

I feel euphoric/high

My muscles relax

Decreased nausea

Increased energy

I have no positive benefits from CBD

Other (please specify)

17. What side-effects do you have from CBD? (Choose all that apply)

Dry mouth

Dizziness

Headache

Fatigue

Vomitting

Nausea

Diarrhoea

Rapid heartbeat

Upset stomach

Problems urinating

Pain

Fainting

Irritability or agitation

Sleep problems

Liver problems (in blood test)

Sexual problems (e.g. erectile dysfunction, reduced libido/pleasure)

Psychotic symptoms (e.g. hallucinations, paranoia, delusions)

Seizures

Anxiety, feeling uneasy

Difficulty concentrating

I don't have any side-effects

Other (please specify)

Other (please specify)

18. Where do you get your CBD from?

From a CBD shop

From a health shop

From a pharmacy

From an online CBD shop (legal)

From the dark-web (illegal)

From a health practitioner who sells CBD

From a social media distributor

From a medical prescriber (physician)

I make it myself

From a dispensary

From a cannabis social club

Other

19. Where do you live?

20. If you are a former CBD user, why did you stop? (Choose all that apply)

It did not help my symptoms

It is too expensive

My doctor told me to stop

I don't need it anymore

I use another medicine instead

I did not like the side-effects

It isn't legal where I live

Because I don't trust the content (unregulated market)

Other (please specify)
