# Supplementary material for: Reasons for cannabidiol use: a cross-sectional study of CBD users, focusing on self-perceived stress, anxiety, and sleep problems
Source: J Cannabis Res. 2021 Feb 18;3:5. doi: 10.1186/s42238-021-00061-5 (PMC7893882; doi:10.1186/s42238-021-00061-5)
Supplement: Supplementary file 1 — Additional file 1: User Survey. Table S1. Demographic variables of the 28 non-responders. Table S2. Reasons for use of cannabidiol by sex, age and location. Results are presented as n (%). [file 42238_2021_61_MOESM1_ESM.zip › Supplementary.materials.2docx.docx]

**Title:** Reasons for cannabidiol (CBD) use: A cross-sectional study of CBD users, focusing on self-perceived stress, anxiety, and sleep problems

**Julie Moltke^1^*, Chandni Hindocha^2,3,4^**

**^1^** Clinic Horsted, Chronic Pain and Medical clinic, Farvegade 2, 1463 Copenhagen, Denmark

^2^ Clinical Psychopharmacology Unit, Department of Clinical, Educational & Health Psychology, , University College London

^3^ Translational Psychiatry Research Group, Research Department of Mental Health Neuroscience, Division of Psychiatry, Faculty of Brain Sciences, University College London

^4^ University College Hospital National Institute of Health Research (NIHR) Biomedical Research Centre

*Author to whom correspondence should be addressed: Julie Moltke, drjuliemoltke@gmail.com, Clinic Horsted, Chronic Pain and Medical clinic, Farvegade 2, 1463 Copenhagen, Denmark

**Short title**: Self-reported effects of CBD on mental health outcomes.

**Word count:**

Abstract: 325

Main text: 5564

**Analysis of Non responders**

There were 28 non-users responding the survey. Examining the responses of non-users show most responses to be missing. Demographics of these individuals show no divergence from the CBD-using population who responded to the survey.

**Supplementary table 1: Demographic variables of the 28 non-responders**

| N=28 | Valid percentage (%) |
| --- | --- |
| Sex (% F) | 60.7 |
| Age (n=28) |  |
| 18-24 | 3.6 |
| 25-34 | 14.3 |
| 35-44 | 28.6 |
| 45-54 | 28.6 |
| 55-64 | 14.3 |
| 65+ | 10.7 |
| Country (n=22) |  |
| UK | 90.9 |
| Other | 9.1 |
| Other Prescription Drug use (n=8) |  |
| Antidepressants/Anxiolytics | 5 |
| Vitamins/supplements | 3 |

**Supplementary table 2: Reasons for use of cannabidiol by sex, age and location. Results are presented as n(%).**

|  | Sex | | Age | | | Location | |
| --- | --- | --- | --- | --- | --- | --- | --- |
| Reason for use | **Males (n=163)** | **Females (n=255)** | **<34 years old (n=116)** | **35-54 years old (n=202)** | **55+ years old (n=98)** | **UK (n=315)** | **Other (104)** |
| For general health and wellbeing | 74(45.4) | 75 (29.4) | 54 (46.6) | 66 (32.7) | 29 (29.6) | 99 (31.4) | 50 (48.1) |
| Stress | 52 (31.9 | 98 (38.4) | 58 (50) | 73 (36.1) | 19 (19.4) | 113 (35.9) | 37 (35.6) |
| Chronic Pain | 33 (20.2) | 57 (22.4) | 17 (14.7) | 50 (24.8) | 21 (21.4) | 63 (20) | 27 (26) |
| Anxiety | 55 (33.7) | 119 (46.7) | 68 (58.6) | 86 (42.6) | 20 (20.4 | 138 (43.8) | 36 (36.4) |
| Insomnia | 27 (16.6) | 68 (26.7) | 27 (23.3) | 42 (20.8) | 26 (26.5) | 73 (23.2) | 22 (21.2) |
| Arthritis/Joint Pain | 30 (18.4) | 52 (20.4) | 9 (7.8) | 31 (15.3) | 40 (40.8) | 63 (20) | 19 (18.3) |
| Depression | 22 (13.5) | 46 (18) | 18 (15.5) | 42 (20.8) | 8 (8.2) | 52 (16.5) | 16 (15.4) |
| PTSD | 3 (1.8) | 16 (6.3) | 6 (5.2) | 9 (4.5 | 4 (4.1) | 16 (5.1) | 3 (2.9) |
| Menstrual Pain | - | 35 (13.7) | 19 (16.4) | 16 (7.9) | 0 | 26 (8.3) | 9 (8.7) |
| Endometriosis | - | 8 (3.1) | 2 (1.7) | 5 (2.5) | 1 (1) | 6 (1.9) | 2 (1.9) |
| Fibromyalgia | 3 (1.8) | 27 (10.6) | 5 (4.3) | 20 (9.9) | 5 (5.1) | 24 (7.6) | 6 (5.8) |
| Parkinson's | 1 (0.6) | 1 (0.4) | 0 | 1 (0.5) | 1 (1) | 0 | 2 (1.9) |
| Alzheimer's | 1 (0.6) | 0 | 0 | 1 (0.5) | 0 | 0 | 1 (1.0) |
| ADHD | 6 (3.7) | 5 (2) | 4 (3.4) | 6 (3.0) | 1 (1) | 8 (2.5) | 2 (2.9) |
| Autism | 1 (0.6) | 2 (0.8) | 1 (0.9) | 2 (1.0) | 0 | 3 (1.0) | 0 |
| Multiple Sclerosis | 1 (0.6) | 3 (1.2) | 0 | 2 (1.0) | 2 (2.0) | 3 (1.0) | 1 (1.0) |
| Huntington's | 0 | 1 (0.4) | 0 | 1 (0.5) | 0 | 1 (0.3) | 0 |
| Epilepsy | 3 (1.8) | 4 (1.6) | 3 (2.6) | 4 (2) | 0 | 4(1.3) | 3 (2.9) |
| Skin conditions | 13 (8) | 22 (8.6) | 17 (14.7) | 12 (5.9) | 6 (6.1) | 23 (7.3) | 12 (11.5) |
| Headaches/migraine | 8 (4.9) | 22 (10.2) | 12 (10.3) | 18 (8.9) | 4(4.1) | 26 (8.3) | 8 (7.7) |
| Asthma or Allergy | 3 (1.8) | 8 (3.1) | 2 (1.7) | 4(2.0) | 5 (5.1) | 8 (2.5) | 3 (2.9) |
| Cancer | 0 | 1 (0.4) | 0 | 0 | 1 (1.0) | 1 (0.3) | 0 |
| Nausea | 0 | 6 (2.4) | 4 (3.4) | 2(1.0) | 0 | 6 (1.9) | 0 |
| To counteract THC | 6 (3.7) | 11 (4.3) | 8 (6.9) | 8 (4.0) | 1 (1.0) | 7 (2.2) | 10 (9.6) |
| To increase focus and productivity | 18 (11) | 21 (8.2) | 18 (15.5) | 17 (8.4) | 4 (4.1) | 26 (8.3) | 13 (12.5) |
| To help improve sleep | 51 (31.3) | 78 (30.6) | 47 (40.5) | 60 (29.7) | 22 (22.4) | 97 (30.8) | 32 (30.8) |
| Post work-out for sore muscles | 23 (14.1) | 18 (7.1) | 18 (15.5) | 18 (8.9) | 5 (5.1) | 26 (8.3) | 15 (14.4) |
| To help reduce restless legs | 11 (6.7) | 13 (5.1) | 7 (6.0) | 12 (5.9) | 5 (5.1) | 18 (5.7) | 6 (5.8) |
